# Characterization of the rat cerebrospinal fluid proteome following acute cerebral ischemia using an aptamer-based proteomic technology

**DOI:** 10.1038/s41598-018-26237-3

**Published:** 2018-05-21

**Authors:** Alba Simats, Teresa García-Berrocoso, Laura Ramiro, Dolors Giralt, Natalia Gill, Anna Penalba, Alejandro Bustamante, Anna Rosell, Joan Montaner

**Affiliations:** Neurovascular Research Laboratory, Vall d’Hebron Institute of Research (VHIR), Universitat Autònoma de Barcelona, Barcelona, Spain

## Abstract

The limited accessibility to the brain has turned the cerebrospinal fluid (CSF) into a valuable source that may contribute to the complete understanding of the stroke pathophysiology. Here we have described the CSF proteome in the hyper-acute phase of cerebral ischemia by performing an aptamer-based proteomic assay (SOMAscan) in CSF samples collected before and 30 min after male Wistar rats had undergone a 90 min Middle Cerebral Artery Occlusion (MCAO) or sham-surgery. Proteomic results indicated that cerebral ischemia acutely increased the CSF levels of 716 proteins, mostly overrepresented in leukocyte chemotaxis and neuronal death processes. Seven promising candidates were further evaluated in rat plasma and brain (CKB, CaMK2A, CaMK2B, CaMK2D, PDXP, AREG, CMPK). The 3 CaMK2 family-members and CMPK early decreased in the infarcted brain area and, together with AREG, co-localized with neurons. Conversely, CKB levels remained consistent after the insult and specifically matched with astrocytes. Further exploration of these candidates in human plasma revealed the potential of CKB and CMPK to diagnose stroke, while CaMK2B and CMPK resulted feasible biomarkers of functional stroke outcome. Our findings provided insights into the CSF proteome following cerebral ischemia and identified new outstanding proteins that might be further considered as potential biomarkers of stroke.

## Introduction

Stroke is one of the most frequent causes of mortality worldwide and is still considered the leading cause of permanent adult disability in developed countries^1^. Despite major improvements have been achieved in the management of stroke patients in regards to acute therapies^2,3^, there is still a need for improving the diagnosis, prognosis and the treatment of patients that had suffered from this devastating disease. To that end, a complete understanding of the key molecular changes that happen as a consequence of the reduction of glucose and oxygen supply to the brain is necessarily required, and has been a matter of study during the last decade for many experts on this field^4^.

Due to limited accessibility to the brain, the study of these cellular and molecular alterations remains laborious. It is not surprising, then, that remarkable efforts have been directed to the systemic identification and evaluation of molecular mediators of ischemic injury that accurately reflect the stroke pathology and its severity. Remarkably, due to the exchange of molecules between brain and blood, circulating biomarkers have been the first line of research thanks to the simplicity and the minimally-invasive blood collection procedure. Blood evaluation has permitted the identification of potential mediators of the stroke pathophysiology in terms of diagnosis and prognosis, despite none of them has reached its clinical implementation yet^5,6^. However, because the brain is strictly protected from circulation by the brain-blood barrier (BBB) and considering that there is still a lack of complete understanding of how different brain molecules reach the peripheral circulatory system, blood biomarkers research has not yet provided enough information to fully understand all the alterations that occur in the brain following stroke.

To overcome these issues, the exhaustive study of other biological samples, such as the cerebrospinal fluid (CSF), which is in much more close contact with the brain than the peripheral circulation, has emerged as a complementary approach for the characterization of stroke pathophysiology. As known for other neurological diseases, this brain protective and supportive body-fluid provides an excellent opportunity to evaluate the very early signs of neuronal degeneration^7,8^. In the case of ischemic stroke, however, the clinical usefulness of CSF for an early diagnosis of stroke is challenging, since collection methods require an invasive procedure that might still be difficult to be perform in clinical situations of emergency and supposes an absolute contraindication for intravenous thrombolytic therapies. Nevertheless, CSF evaluation in the field of ischemic stroke might provide additional insights about the pathogenic alterations underlying cerebral ischemia, and may contribute to the complete interpretation of stroke pathology and the identification of potential therapeutic targets for its pharmacological modulation^9^. In this context, the use of pre-clinical models has proven to be notably relevant to examine those biological fluids that are difficult to obtain from human, as for the case of CSF.

The emerging proteomic technologies provide new high-throughput approaches that allow the simultaneous identification of a huge quantity of proteins in biological samples, including fluids, especially serum or plasma, and, although to a lesser extent, post-mortem brain tissue of ischemic stroke subjects^10^. Due to the wide range of protein abundance in the circulating proteome, however, the identification of low abundant brain-specific molecules in body fluids is frequently a limitation^11^. To overcome this challenge, continuous advances in these proteomics’ approaches are being made. Concretely, the SOMAscan proteomic assay, which is based on the usage of modified nucleic acid aptamers (named SOMAmers: Slow Off-rate Modified Aptamers), facilitates the simultaneous identification of an extensive set of proteins across a wide range of concentrations and abundances by replacing direct protein measurement into a straightforward DNA quantification, independently of high abundant protein confounders^12^.

In this study, our aim was to describe the CSF proteomic profile at a very early stage after cerebral ischemia in rats by using the SOMAscan proteomic technology. Moreover, we further explored our results to deeply describe seven selected candidates with a potential involvement in the stroke pathophysiology. To that end, these molecules were examined in blood and brain tissue of ischemic and sham-control animals and were tested in stroke patients for their ability as diagnosis and prognosis biomarkers.

## Results

### Proteomic profiling of CSF after cerebral ischemia

**Figure 1:**
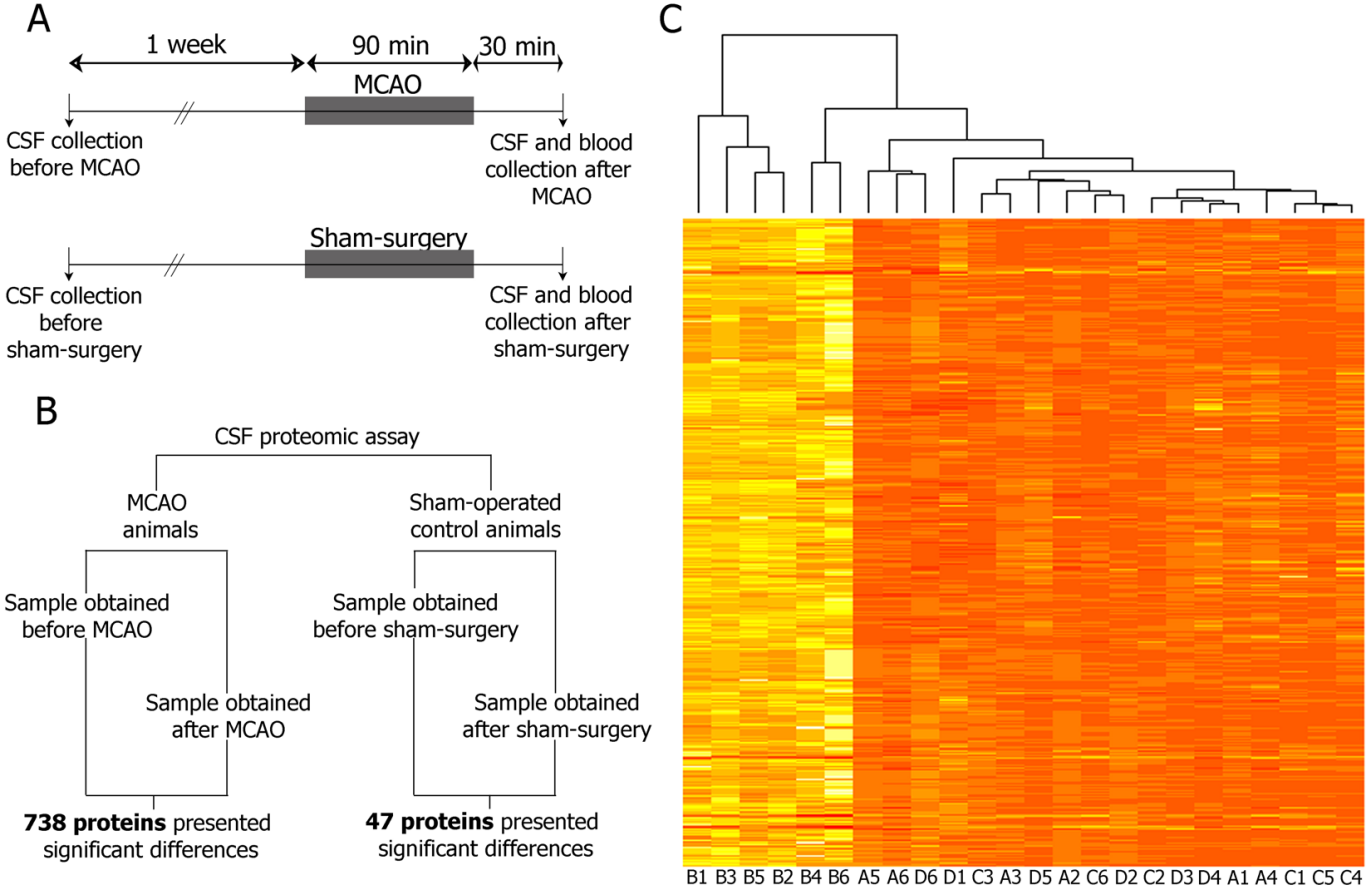
(**A**) Schematic representation of the design of the experimental study. (**B**) Schematic representation of data analyzed by the SOMAscan proteomic assay. (**C**) Color map of relative fluorescence units (RFU) of the 738 proteins that were significantly altered after the ischemia. Squares are color coded; bright yellow color indicates high RFU and pale orange color indicate low RFU. The brighter the color is, the higher RFUs are. A1-A6 indicate samples obtained before MCAO; B1-B6 indicate samples obtained 2 h after MCAO; C1-C6 indicate samples obtained before sham surgery; D1-D6 indicate samples obtained after sham surgery. Abbreviations: CSF: cerebrospinal fluid; MCAO: middle cerebral artery occlusion.

To characterize the CSF proteomic profile in the hyperacute phase of cerebral ischemia, we harvested CSF from MCAO and sham-control animals at two different time-points: before (pre) and after (post) their respective surgeries (Fig. 1B). No tangible differences in relative protein abundances were detected in CSF samples from sham-control animals, with only 47 proteins (4.16%) differentially regulated in CSF between the pre and post sham-control surgical procedure. Interestingly, a large amount of proteins (738; 65.4% of total) increased in rat CSF after the induction of cerebral ischemia compared to their pre samples (Fig. 1C and Supplementary Table S1).

**Figure 2:**
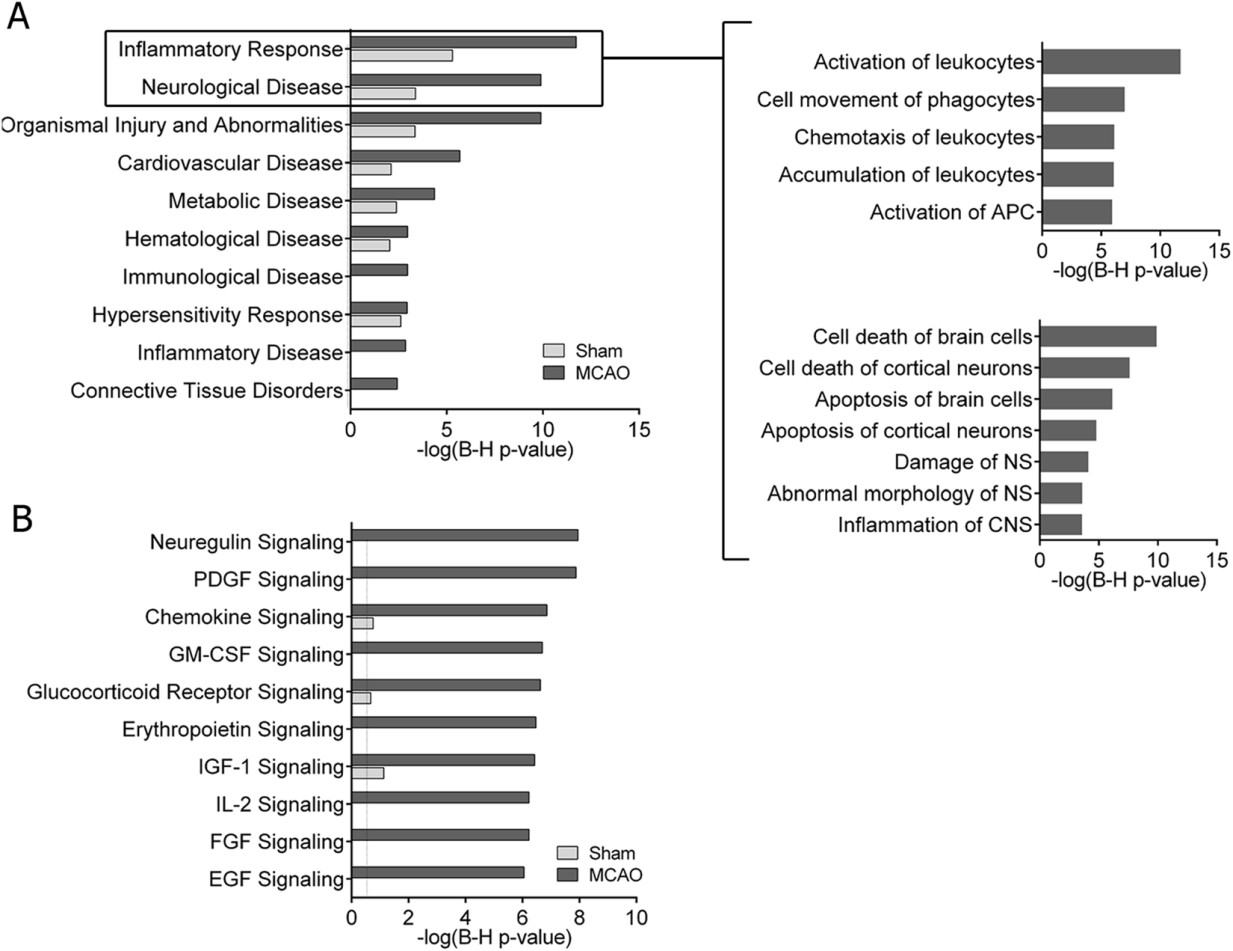
IPA analysis of differentially expressed proteins in CSF after cerebral ischemia in rats. Analyses were classified by disease and disorders (**A**) and canonical pathways (**B**) and compared with the CSF from the control group of sham-operated animals. Magnification of the top 2 canonical pathways from A is also provided. Top 10 categories ranked by MCAO corrected p-value are listed in all graphs.

CSF differentially-expressed proteins after MCAO and sham-control surgery were further characterized through bioinformatics analysis. The disease- and function-related analysis (Fig. 2A) revealed that the inflammatory response that early appears in the brain after an ischemic event was the most overrepresented biological process in the CSF proteome 2 h after cerebral ischemia. Specifically, the activation and accumulation of leukocytes, and several inflammatory processes related to the infiltration of immune cells into the brain, including immune cells movement and leukocytes chemotaxis, were largely represented in the CSF from MCAO animals. Furthermore, particular interest was also directed towards the specific biological processes encompassed in the category of neurological disease, since molecular mechanisms of cell death of brain cells, specifically of cortical neurons, were the foremost annotations reported in the CSF at this early time-point after ischemia (Fig. 2A).

Notably, the analysis of the main canonical pathways revealed that the neuroregulin and the platelet-derived growth factor (PDGF) molecular pathways were also highlighted processes in the acute phase of ischemia, as reflected by their highlighted relevance in the CSF proteome exclusively from ischemic animals (-log(B-H p-value) > 7). Moreover, additional strength was given to inflammation in the early phase of ischemia, since chemokine signaling pathways were also highly represented in the CSF proteome profile after cerebral ischemia (Fig. 2B).

### Selection of proteins with significantly altered expression in CSF after cerebral ischemia

Twenty-two out of the 738 differentially-expressed proteins after MCAO (2.98%) were also up-regulated after sham-control surgery, namely common in both dataset comparisons. Since our attention was focused on the key proteomic alterations after cerebral ischemia, and in order to minimize the influence of the non-ischemic processes associated to the surgery procedure, these 22 common proteins were discarded for further analysis. Therefore, we finally identified 716 proteins that were exclusively up-regulated due to brain ischemia.

Of those stroke-associated proteins, 46 (6.42%) presented more than a 4-fold up-regulation in the CSF after MCAO compared to pre MCAO (the top 20 proteins are shown in Table 1).

**Table 1:** Top 20 proteins increased in the CSF after MCAO.

Protein name	Acronym	Pre vs. Post MCAO	
		FDR p-value	Log(FC)
**Creatin kinase B-type**	**CKB**	**0.0393**	**3.4370**
**Calcium/Calmodulin dependent protein kinase II subunit beta**	**CAMK2B**	**0.0393**	**2.7742**
**Pyridoxal phosphate phosphatase**	**PDXP**	**0.0393**	**2.7283**
**Amphiregulin**	**AREG**	**0.0393**	**2.6482**
**Calcium/Calmodulin dependent protein kinase II subunit delta**	**CAMK2D**	**0.0393**	**2.6411**
Junctional adhesion molecule C	JAM3	0.0393	2.5815
Cytokine receptor common subunit gamma	IL2RG	0.0393	2.5596
40S ribosomal protein S7	RPS7	0.0102	2.5538
Fibroblast growth factor 16	FGF16	0.0393	2.5361
Ubiquitin-conjugating enzyme E2 L3	UBE2L3	0.0393	2.5222
Interleukin-23 receptor	IL23R	0.0393	2.5061
Vacuolar protein sorting-associated protein VTA1	VTA1	0.0183	2.5013
**Calcium/Calmodulin dependent protein kinase II subunit alpha**	**CAMK2A**	**0.0393**	**2.5007**
Seizure 6-like protein 2	SEZ6L2	0.0393	2.4662
Glycoprotein hormones alpha chain/Lutropin subunit beta	CGALHB	0.0393	2.4515
Protein kinase C iota type	PRKCI	0.0393	2.4042
NKG2D ligand 3	ULBP3	0.0393	2.3961
Desert hedgehog protein	DHH	0.0393	2.3926
Fibroblast growth factor 7	FGF7	0.0393	2.3710
Apoptosis regulator Bcl-2	BCL2	0.0393	2.3698

Proteins selected for further examination are highlighted in bold.

**Figure 3:**
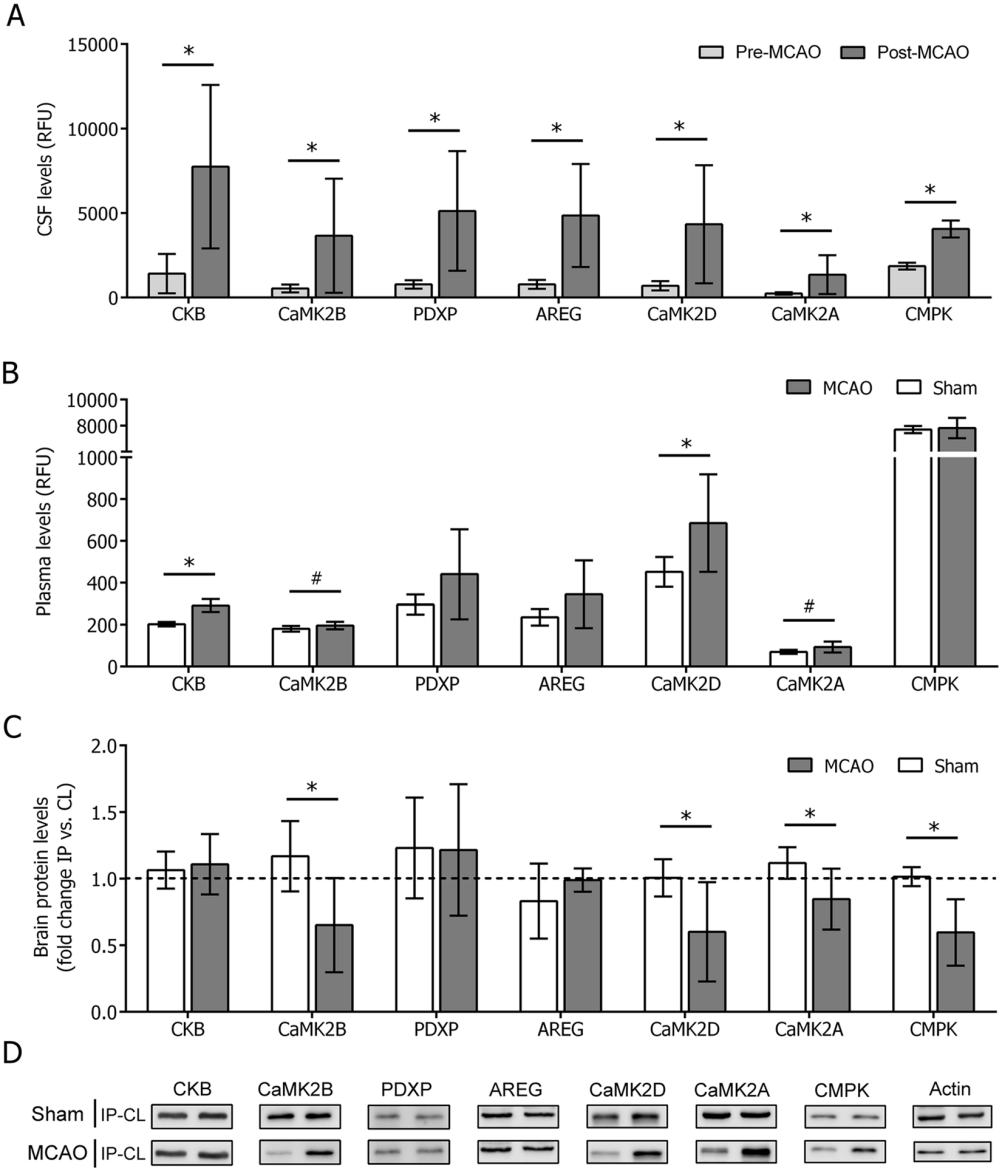
Exploration of protein levels in CSF, plasma and brain of ischemic and sham-control animals. Plots of protein abundance data (RFU: relative fluorescent units) in (**A**) CSF samples obtained pre- and post-MCAO and (**B**) plasma samples obtained 2 h after MCAO or sham-control surgery (n = 6 samples per experimental group for all cases). (**C**) Plot of protein abundance (relative quantification, ratio IP vs. CL hemisphere of each animal) in brain homogenates samples obtained 2 h after MCAO or sham-control surgery (n = 4 samples for sham; n = 8 samples for MCAO). In all cases, IP and CL samples from each animal are run in the same gel and individually corrected by their respective β-actin loading controls (Supplemental Figure S3). (**D**) Representative Western Blots signals used for quantification of proteins in brain. Bars indicate mean ± SD. For all graphs, *p < 0.05 and ^#^p < 0.1. Abbreviations: IP: ipsilateral hemisphere; CL: contralateral hemisphere.

For further studies, the top five proteins from this list (CKB, CaMK2B, CaMK2D, PDXP and AREG) were chosen as promising candidates to be examined in detail. Calcium/Calmodulin-dependent protein kinase II subunit alpha (CaMK2A) was additionally chosen for further exploration, given the outstanding importance of their analogues (CaMK2B and CaMK2D), and Uridine monophosphate/cytidine monophosphate (UMP/CMP) kinase (CMPK) protein was also included as a candidate due to its relevance in a previous study from our group (FDR p = 0.008, logFC = 1.127)^13^ (Fig. 3A). None of the selected candidates showed an association with the acute neurological deficits of ischemic animals (data not shown).

### Analysis of the selected proteins in the rat brain and blood

The proteomic profile of plasma samples, which were also obtained 2 h post MCAO or sham-control surgery from the same set of animals, was run in parallel. Of all selected candidates, only CKB and the 3 members of the CaMK2 protein family showed an increase in plasma of MCAO animals compared to sham controls (Fig. 3B). Additionally, these higher circulating levels were positively correlated with the CSF levels for the CaMK2 members (R = 0.736, p = 0.006 for CaMK2B; R = 0.589, p = 0.044 for CaMK2D; and R = 0.626, p = 0.029 for CaMK2A), whereas no correlation between plasma and CSF levels post MCAO was seen for CKB (R = 0.161, p = 0.617). Conversely, neither PDXP nor AREG or CMPK showed differences in their circulating plasma levels between MCAO and sham-control animals at the studied time-point, and neither of them showed correlation between plasma and CSF levels post MCAO.

Protein candidates were further characterized in brain homogenates from the same set of MCAO and sham-control animals. No appreciable differences were detected for PDXP, AREG and CKB levels between the affected and healthy brain hemispheres of MCAO animals and sham controls, as reported by the ratio between ipsilateral (IP) and contralateral (CL) protein levels (p = 0.956, p = 0.190 and p = 0.736, respectively). However, CMPK and the three members of the CaMK2 family (CaMK2B, CaMK2D and CaMK2A) did show a decrease in their levels in the ischemic hemisphere compared to the healthy side of the brain and sham controls (p = 0.032, p = 0.075, p = 0.037 and p = 0.011, respectively) (Fig. 3C,D). Interestingly, the simultaneous evaluation of the total levels of CaMK2 (phosphorylated and non-phosphorylated forms) resulted in no differences between MCAO and sham-control animals (p = 0.817), whereas the detection of the phosphorylated form alone did show a slight increase in the infarcted hemisphere compared to the contralateral in ischemic animals (p = 0.090) (Supplemental Figure S2).

**Figure 4:**
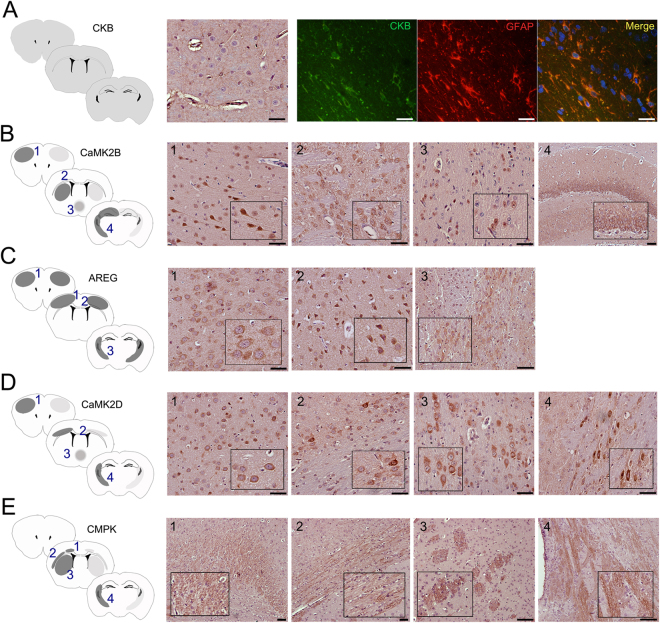
Histological localization of protein candidates in the ischemic brain. (**A**) CKB, (**B**) CaMK2B, (**C**) AREG, (**D**) CaMK2D and (**E**) CMPK histological examination in three established cortical depths of the ischemic brain (see Supplemental Figure S1). In the schematic representation of brain, right hemisphere corresponds to IP and left hemisphere corresponds to CL. Dark grey regions indicate higher abundance of positive staining than pale gray zones. CKB (green) was co-localized with GFAP marker in the red channel and nuclear detection was conducted with DAPI in the blue channel. Scale bar = 50 µm.

The specific brain localization in ischemic rats was conducted for each selected protein with commercially-available antibodies for immunohistochemistry. Relative abundances between IP and CL hemispheres were consistent with the aforementioned results for all candidates. CKB immunostaining was consistent through all brain regions and co-localized with the astrocytic marker GFAP (Glial Fibrillary Acidic Protein) (Fig. 4A), but not with the microglial marker Iba-1 (data not shown). CaMK2B positive staining was observed in neurons from the pyramidal layers of the cerebral cortex and in striatal neurons. Moderate staining of CaMK2B was also shown in hypothalamic nuclei from the periventricular region and in the hippocampus (Fig. 4B). Staining of AREG was detected in both, pyramidal and granular neuronal layers. Moreover, AREG-positive signal was also seen in the third coronal brain depth and was localized just below the ending lateral ventricles, close to the reticular nucleus of the thalamus (Fig. 4C). CaMK2D positive staining was shown in cortical neurons from the granular layer IV and the multiform layer VI, it was also identified in the periventricular region of the hypothalamus, similar to its analogue CaMK2B, and presented robust positive staining in neurons from the internal capsule of the corticospinal tract (Fig. 4D). Finally, CMPK staining was observed in areas close to the *cingulum*, external and internal capsule, and was notably pronounced in neuronal bundles from the striatum (Fig. 4E).

### Translation to potential biomarkers in stroke patients

We further aimed to assess the potential role of CKB, CaMK2B, CaMK2D, CaMK2A and CMPK as biomarkers of stroke. With that purpose, we evaluated circulating levels of these proteins in human blood samples from ischemic stroke (<6 h from symptoms onset) and control subjects. Demographical and clinical characteristics of all patients are described in Supplemental Table S2. In brief, ischemic stroke subjects were older and presented higher prevalence of atrial fibrillation and ischemic cardiopathy than control individuals. However, differences in the circulating levels of the selected candidates could not be explained by any of these demographical and clinical variations (data not shown).

**Figure 5:**
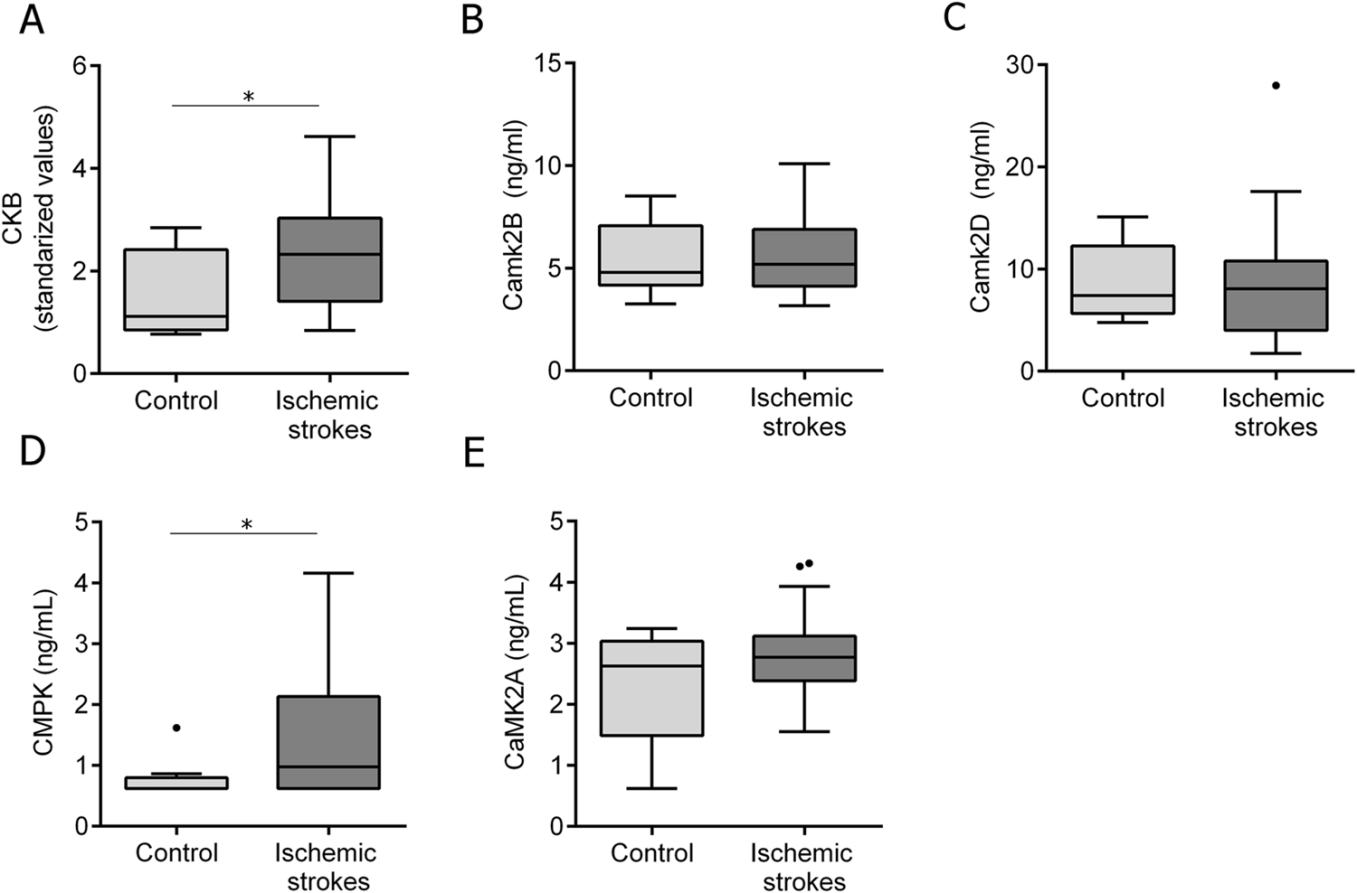
Plasma levels of CKB, CaMK2-family members and CMPK. Circulating levels in ischemic stroke patients (n = 38) and controls (n = 8) of all selected candidates. Blood samples were obtained on patient admission to the hospital (<6 h from symptoms onset). Median and Tukey confidence intervals are represented in each graph. Standardized values for CKB were calculated using Z-score (see Supplemental materials and methods data). For all graphs, *p < 0.05.

Notably, circulating levels of CKB and CMPK were higher in ischemic strokes than controls (p = 0.050 and p = 0.047, respectively) (Fig. 5A,D). However, neither CaMK2B nor CaMK2D or CaMK2A showed differences in their circulating levels between both studied groups (p = 0.719, p = 0.904 and p = 0.687, respectively) (Fig. 5B,C and E).

Next, we aimed to evaluate the practical use of these proteins as biomarkers of long-term functional outcome after stroke. Seventeen out of 38 ischemic stroke patients (44.7%) exhibited a poor functional outcome by the third month after stroke. Notably, baseline circulating levels of CaMK2B and CMPK were higher in those patients with poor functional outcome than patients with good functional outcome (6.23 ± 1.96 ng/mL vs. 5.02 ± 1.41 ng/mL, p = 0.046 for CaMK2B; 1.43 [0.9–2.39] ng/mL vs. 0.62 [0.62–1.09] ng/mL, p = 0.019 for CMPK), but did not associate with stroke severity, as measured by the NIHSS at admission (p = 0.734 and p = 0.773 respectively).

We found a cut-off point of 5.016 ng/mL for CaMK2B (70% sensibility, 69% specificity) and 0.759 ng/mL for CMPK (76% sensibility, 71% specificity) that were significantly associated with poor functional outcome at the studied time-point (p = 0.020 and p = 0.014, respectively). The predictive clinical model including the variables significantly associated with poor outcome in the univariate analysis (Supplemental Table S3) was further adjusted by age, sex and NIHSS score at admission, which are well-known factors that highly influence stroke prognosis^14^. The addition of each biomarker individually to this predictive model showed that both of them remained independent predictors of poor outcome (OR = 9.82 [1.13–85.88], p = 0.039 for CaMK2B; OR = 9.40 [1.53–57.67], p = 0.015 for CMPK). The combination of both biomarkers in the same predictive model was also an independent predictor of poor outcome (OR = 6.38 [1.33–30.69], p = 0.020).

## Discussion

The study being presented here aimed to map the rat cerebrospinal fluid proteome in the hyperacute phase of cerebral ischemia by using an aptamer-based proteomic technology as a research tool to identify potential stroke biomarkers. The SOMAscan approach facilitated the screening of hundreds of proteins in CSF, and permitted the evaluation and characterization of its protein composition following an event of cerebral ischemia.

The cerebrospinal fluid is the physiological medium that surrounds and mechanically supports the central nervous system (CNS). The CSF circulates unidirectionally from the brain ventricles to the subarachnoid space in the cortex and serves as a transporter of biochemical messages from one brain region to another. Approximately an 80% of the CSF originates from the filtration of arterial blood in the choroid plexus, whereas the rest is thought to principally derive of the drainage of brain interstitial fluid (ISF)^15,16^. The connection between CSF and ISF is strictly necessary for an optimal maintenance of the neuronal microenvironment in the brain parenchyma. Under cytotoxic conditions, ISF conducts metabolic waste to clear away cellular degradation products, leading to an accumulation of catabolites diffusing out from brain to the CSF^17^. Following an ischemic event, unique signatures of brain-specific molecules from damaged parenchyma come together in the CSF. The early accumulation of these brain-derived factors in the CSF might accurately reflect the current status of the brain, and its collection might in turn facilitate a rapid diagnosis of the on-going CNS pathological processes and accelerate therapeutic interventions as required.

Bioinformatics’ analysis of the CSF composition after cerebral ischemia reinforced the well-known importance of the inflammatory response in the ischemic brain^18^. After only 2 hours from ischemia onset, the signaling cascade of inflammation was well established and molecularly reflected in the CSF. Concretely, mediators of the mobilization of activated leukocytes to the ischemic site of injury, including proteins involved in cellular movement and chemotactic factors, were of considerable relevance in the ischemic CSF proteome. The diffusion into the CSF of proteins that participate in pathways of neuronal cell death was also found to be of great magnitude at the studied time-point. As it is well-known, the breakdown of neuronal cell integrity causes a devastating loss of neurons in the core of the ischemic lesion within a short period of time from disease onset. The identification and functional characterization of mediators of (or induced by) this rapid stroke-related massive injury might serve as valuable therapeutic targets for the reduction of the brain lesion. This is the case of the neuroregulin and PDGF pathways, since both signaling pathways have been related to neuroprotection: neuroregulin has shown potential therapeutic value by preventing macrophage/monocytes infiltration and astrocyte activation^19^, whereas PDGF has been proposed to be angiogenic in stroke^20^.

The top seven outstanding proteins found at high levels in the CSF following cerebral ischemia in the present study have been extensively described in the context of the stroke pathophysiology. CK-BB, composed by two identical CKB subunits, is the most abundant creatine kinase (CK) isoenzyme in the brain. CK isoforms play a key role in energy transduction and homeostasis, especially in high-demanding tissues, by catalyzing the reversible exchange between creatine and creatine-phosphate, thus facilitating the conversion of ADP to ATP and vice versa to cover cell energy requirements and storage^21^. CK in the isoforms of CK-MM and CK-MB have been widely studied for the diagnosis of myocardial infarction^22^, but less is known about CK-BB disease relevance. In concordance with our results, CK-BB has been well reported to immediately increase in blood circulation and CSF following acute cerebral injuries, including cerebrovascular traumatisms, meningitis and strokes^23–25^, and its role as a plausible diagnostic biomarker for ischemic stroke has been previously postulated^26,27^. CK-BB provenance has been slightly controversial; some studies have proven that astrocytes and neurons are the main CK-BB source, whereas others, including us, could only proof that astrocytes express CK-BB in brain^28^. Our findings indicate that there is a strong CK-BB accumulation in the CSF and blood following ischemia in rats; despite we were not able to detect differences in CK-BB levels in brain parenchyma between the infarcted and healthy hemispheres of the brain.

AREG (amphiregulin) was another outstanding protein found to have increased levels in CSF after cerebral ischemia. AREG is a member of the epidermal growth factor (EGF) family of proteins. This EGF-like molecule induces cell differentiation and proliferation and contributes to wound healing and tissue repair following infection or injury^29^. In brain, AREG remarkably increases in response to ischemic conditions in cortical, striatal and hippocampal neurons, and attenuates neuronal damage by inhibiting the pro-inflammatory-related endoplasmic reticulum stress^30^. In the present study, AREG markedly accumulate in the CSF of ischemic animals but their plasma and in-brain levels did not change after the insult. This controversy might be explained in part by a direct balance between a higher intracellular release caused by the on-going massive cell disruption and death in the ischemic core, and a simultaneously higher expression of this protein in an attempt to slow-down and retrain stroke-related neuronal damage. Besides, AREG has been also linked to the propensity to develop hemorrhages in thrombolytic-treated ischemic stroke patients^31^. Concretely, AREG increases the expression of MMP-9 and VEGF, both molecules strongly associated with hemorrhagic complications after stroke^32,33^. Thus, although the time-point studied here precludes the study of hemorrhagic complications and their association with AREG levels, further experiments should be conducted to explore it as a therapeutic target.

PDXP, also named as chronophin, regulates the synthesis of pyroxidal 5′-phosphate (PLP), the coenzymatically active form of vitamin B6. It removes the phosphate group from PLP and degrades it to 4-pyridoxic acid^34^. PDXP participates in the biosynthesis of various neurotransmitters and organic molecules, and is found at high levels in brain and testis^35,36^. PDXP is also an important regulator of cofilin or actin-depolymerization factor (ADF)^37^. ADF/cofilin maintains a regular intracellular reservoir of ATP-G-actin monomers by the depolymerization of older ADP-F-actin, thus regulating actin dynamics and contributing to the construction and remodeling of a great variety of polarized structures within cells^38^. Activated ADF/cofilin (non-phosphorylated form) has been shown to mediate apoptotic processes, and its inhibition (phosphorylated form) has shown promising neuroprotective results by increasing neuronal viability and survival^39^. PDXP is known to activate ADF/Cofilin, thus further contributing to mechanisms of neuronal damage and death^40^. PDXP itself is activated in response to different intracellular and extracellular signals of stress, such as oxidative stress or the drop of intracellular ATP that occurs after cerebral blood flow impairment^34,37^. PDXP levels increase in brain following cerebral ischemia, and decrease after the administration of neuroprotective free radical scavengers, such as ferulic acid^34,41^. In our study, we reported high levels of PDXP in CSF early after ischemia, but we were not able to detect changes in brain levels neither in plasma at the studied time-point. It remains to be tested whether PDXP increases in the ischemic brain at later stages after ischemia, which would potentially serve as a therapeutic target to mediate the mechanisms of stroke-related tissue injury.

CMPK reversibly catalyzes the addition of a phosphate group to UMP and CMP to consequently form UDP and CDP, which are further converted to their triphosphorylated form to be used as substrates of the DNA and RNA polymerases^42^. In brain, these nucleotides are strictly required for the synthesis of neuronal membrane phospholipids, and thus influence various membrane-dependent processes such as neurotransmitter release and neurite outgrowth^43^. A decrease expression of CMPK has been related to aging^44^, whereas an increased expression has been seen in brain cortex from patients with temporal lobe epilepsy and following sciatic nerve injury in rodents^45,46^. In terms of ischemia, decreased levels of both, nucleotides and CMPK, has been observed in the infarcted region of the brain after transient cerebral ischemia^13,47^, which we further corroborated by also reporting decreased levels of CMPK in the early ischemic rat brain. Besides, at the same time-point following ischemia, we observed an increased accumulation of CMPK in the CSF, which suggest that this lower content of CMPK in the ischemic core could be a direct consequence of a massive release of components from disrupted cells within the injured area, rather than a brain injury-related decrease in CMPK expression. On the other hand, despite we could not observe any increase in CMPK levels in circulation 2 h after ischemia in rats, we reported higher CMPK blood levels in ischemic patients within the first 6 h from symptoms compared to controls. Moreover, we could explore the role of CMPK as marker of stroke patient poor prognosis. As reported in the field of cancer^48^, we observed that circulating levels of CMPK at admission were associated with poor long-term outcome, independently of the patient clinical factors. Taking into consideration the involvement of CMPK in mechanisms to combat cell disruption and neuronal cell death^49^, higher CMPK levels following ischemia could be associated with severe ischemic lesions and a final patient poor outcome. Nevertheless, further studies are needed to verify both, the observed diagnostic and prognostic value of CMPK, and evaluate whether CMPK can also serve as a valuable target for therapeutic strategies.

Finally, CaMK2 members (CaMK2A, CaMK2B and CaMK2D) belong to a group of Ser/Thr protein kinases that has been previously involved in glutamate excitotoxicity-induced neuronal cell death^50^. CaMK2 members are primary activated under ischemic conditions by binding of Ca^2+^/Calmodulin and achieve full activity after autophosphorylation of all former subunits by their neighbors^50^. Phosphorylated CaMK2 remains active and maintains its autonomous activity after dissociation of Ca^2+^/Calmodulin, which facilitates and prolongs CaMK2 functional activity^51^. Glutamate citotoxicity causes translocation of the activated CaMK2 members to the post-synaptic sites and extra-synaptic clusters^52,53^, where are involved in synaptic plasticity and serve as a reservoir of CaMK2 to avoid their massive phosphorilation within cells, respectively. Active CaMK2 members have a controversial role in mediating both apoptotic cell death and cell viability^50^. Some of the CaMK2-downstream targets, including different Ca^2+^ channels, promote neuronal apoptosis by enhancing death-inducing overload of cellular Ca^2+ ^^50^, while the modulation of other factors, such as the inhibition of caspase 2 and Bad or the enhanced expression of Bcl-xL, might also attenuate those apoptotic processes^54,55^. In accordance, both cerebroprotective but also neurotoxic properties of CaMK2 inhibition have been described previously^56–58^. However, more promising results have been reported in terms of neuroprotection by suppressing CaMK2 activity before, during or after the ischemic insult, both *in vitro* and *in vivo*^58–60^. In our study, we described decreased levels of the non-phosphorylated inactive CaMK2 and increased levels of the phosphorylated active CaMK2 in the ischemic brain hemisphere compared to the healthy side of the brain and sham controls, as others have also stated^61^. This early increase in the active forms of CaMK2 might be promoted by their involvement in ischemia-related neural plasticity processes and might also participate in the early on-going apoptotic mechanisms within the ischemic lesion. Moreover, CaMK2 levels accumulated in the CSF early after ischemia, and this increase was also reflected in the peripheral circulatory system of ischemic rats. However, these results did not match with those from the pilot study, where circulating levels of CaMK2 were not increased in patients that had suffered an ischemic stroke, and hampers the use of these proteins as potential blood biomarkers of stroke diagnosis. Notably, CaMK2B, but not CaMK2A neither CaMK2D, appeared as a valuable indicator of stroke patient functional outcome. CaMK2B association to disease prognosis has been previously reported in cancer^62^ but, as far as we know no previous study has explored CaMK2B prognosis-value by measuring its levels in the circulation. Thus, in this preliminary study we are newly describing that circulating levels of CaMK2B showed a potential to predict stroke patients outcome, though larger studies are required to corroborate these findings and to assess whether this predictive capacity is unique to CaMK2B, which is the most brain-specific protein of the CaMK2 members^35^, and whether it turns out to also be a potential target to therapeutically modulate the progression of the ischemic lesion.

Importantly, we could not identify a consistent pattern among brain, CSF and blood levels relation between the studied candidates. The lack of a reliable correlation between these three compartments hampers the fully comprehension of their interrelationship in stroke pathology and leads to presume that there might be several unknown mechanisms that selectively and distinctively regulate the exchange of molecules between one and another compartment following an ischemic event. We propose that further efforts should be made to understand this brain-CSF-blood connection, which would offer a huge breakthrough for the complete understanding of stroke disease.

Our work presents some limitations that should be taken into consideration for further studies. First, although the animal model chosen in this study is highly suitable to evaluate the biological alterations of acute ischemic stroke, some additional injury due to reperfusion cannot be avoided, which can lead to the identification of surrounding processes of ischemic stroke itself that are not found in all clinical scenarios^63^. Second, due to technical incompatibilities, we were not able to determine the lesion volumes in the ischemic animals, which would have also been an interesting parameter to analyze. Besides, the SOMAscan platform used here for candidate discovery is initially designed to detect serum human proteins. Despite a great majority of those human-based aptamers show high reactivity based on homology to their rodent counterparts, we cannot discard the presence of potential confounding proteins identified in our datasets. However, we present further analysis with other techniques and tissues confirming the relevance of some of the selected proteins. Finally, despite our clinical pilot study supports the findings on experimental data, we are aware of its limitation due to the reduced number of patients included. Larger and independent cohorts should be studied to validate these results in a near future.

This work also has some strengths that should be highlighted. This study has originated an extended source of important contributors of stroke pathology, which has been validated through the achieved congruence between the discovery datasets and the further exploration of the selected candidates in brain and blood. In this same line, previously known candidate biomarkers for stroke diagnosis and prognosis, including interleukin 6^64,65^, C-reactive protein^66,67^, CC-chemokine ligand 23^68^ or D-dimer^69,70^, have also been corroborated here, since they were also highly increased in CSF 2 h after stroke onset (data not shown). Hence, this additional information further validates and gives potential effect to our study, which we expect it to serve as a potential tool to elucidate future mediators of stroke pathology, provide other feasible biomarkers of stroke and support further potential discovery studies in the field of cerebral ischemia.

In conclusion, we have used here for the first time a high multiplexed, sensitive and quantitative proteomic tool for mapping the rat cerebrospinal fluid proteome in the hyperacute phase of cerebral ischemia. The SOMAscan proteomic approach has facilitated an unbiased screening of hundred of proteins in CSF and plasma samples from ischemic animals, and has originated an extended source of important contributors of stroke pathology that might be supporting future studies in this field. Through this strategy we have been able to firstly identify and primary evaluate the role of three promising proteins as stroke blood biomarkers, which provides added value on the design and development of the present study.

## Methods

### Animals

All experimental procedures were conducted in compliance with the Spanish legislation and in accordance with the Directives of the European Union and were approved by the Ethics Committee of the Vall d’Hebron Institute of Research (protocol number 58/14). All experiments were conducted in a randomized manner and in adherence to the ARRIVE guidelines. Male Wistar rats were used in the experiments (7–12 weeks; Charles River Laboratories Inc., Wilmington, MA, USA). Animals were kept in a climate-controlled environment on a 12 h light/12 h dark cycle. Food and water were provided *ad libitum*. Analgesia (Buprenorfine, 0.05 mg/kg, s.c, Divasa Farma-Vic S.A, Barcelona, Spain) was administered before surgeries to all animals to minimize pain and discomfort. All animals were anesthetized with isoflurane (4% for induction; 2% for maintenance in air, Abbot Laboratories, Spain) and body temperature was maintained at 37 °C during all surgical procedures.

A total of 41 rats were needed to complete the study: 14 animals were subjected to sham-control surgery and 26 to transient middle cerebral artery occlusion (MCAO). One sham-control animal died during surgery and 10 ischemic animals were excluded after applying the following criteria: inappropriate occlusion or reperfusion of the middle cerebral artery as described below (n = 6), massive surgical bleedings (n = 1) and death during the surgery procedure (n = 3).

### Model of transient MCAO

Cerebral ischemia was induced by mechanical occlusion of territory of the MCA, as previously described^71,72^. In brief, the day before surgery, cranial trepanation was performed to attach a laser-Doppler probe (Moor Instruments, Devon, UK) and monitor regional cerebral blood flow (CBF). The following day, a silicone-coated nylon filament (Doccol Corporation, reference number: 403723PK10) was introduced through the external carotid artery and pushed to the internal carotid artery to occlude the MCA. Animals were allowed to recovery during the MCA occlusion period. Ninety minutes later, animals were re-anesthetized to induce a 30-min reperfusion by removal of the filament. Successful occlusion and reperfusion of the MCA was guaranteed by the reduction or increase in the CBF recorded by the laser Doppler probe. Only animals that exhibited a CSF reduction >75% after filament placement and a recovery of >75% after filament removal were included in the study. Sham-control surgery was performed by the same surgical procedures without insertion of the nylon-coated filament.

### Acute neurological deficit

Rats were assessed using the modified Bederson scale, as described elsewhere^73^. Neurological deficits were evaluated in a blinded manner at 80 minutes of MCAO, before reperfusion.

### CSF collection

CSF was collected from rats one week before (pre) and 2 hours after MCAO or sham-control surgery (post) (Fig. 1A). CSF was collected by using a minimally-invasive method of CSF sampling, previously described by Mahat and colleagues^74^. In brief, animals under anesthesia effects were placed in a stereotactic apparatus and properly fixed to create an angle of approximately 110° between the base of the stereotaxic frame and the animal snout. After localizing the rhomboid depressed region, corresponding to the cisterna magna, a 25G-needle connected to a collection apparatus was vertically inserted and slowly advanced until CSF started flowing into the collection tube. After obtaining the sample, the collection tube was clipped to avoid blood contamination and the needle was withdrawn from the cisterna magna.

If blood contamination occurs at any point during CSF collection, the collection tube was cut off from the point of blood contamination. Only clear CSF without visual sings of blood contamination was collected and frozen at −80 °C until further use.

### Rat brain and blood samples

Immediately after CSF collection blood samples were drawn through transcardiac puncture in EDTA tubes and immediately centrifuged at 3,000 g for 10 minutes at 4 °C to obtain plasma, which was frozen at −80 °C until further use. Animals were then euthanized and transcardially perfused with cold saline. Brains were carefully removed and IP and CL hemispheres were separated. Each hemisphere was individually frozen at −80 °C. Frozen hemispheres were cut into 1 mm coronal slices over dry ice. Slice corresponding to the bregma point, where the infarction territory is located, were selected for all animals, thawed and homogenized using mirVana™ PARIS™ RNA and Native Protein Purification kit (ThermoFisher Scientific Inc., Waltham, MA, USA). Protein homogenates were centrifuged at 12,000 g for 10 min at 4 °C and the cleared protein extract was quantified using bicinchoninic acid (BCA) assay (ThermoFisher Scientific Inc.) and frozen at −80° until further use.

Brains from MCAO animal were also used for immunohistological purposes. Those rats were euthanized and transcardially perfused with cold saline and their brains carefully removed and fixed with formalin. Fixed brains were carefully cut into segments at three precisely controlled depths: (1) 2.1 ± 0.2 mm anterior to bregma, (2) bregma and (3) 1.5 ± 0.2 mm posterior to bregma (Supplementary Figure S1). The formed tissue sections were mounted on blocks and embedded with paraffin. Tissue slices of 3 µm were cut, mounted onto glass slides and kept at 4 °C for the IHC experiments.

### SOMAscan proteomic assay

The SOMAscan assay was performed by SomaLogic Inc. (Boulder, CO, USA). SOMAScan is a relative quantitative proteomics assay based on the usage of SOMAmers, which are modified nucleic acid aptamers that permit the identification and quantification of a library of human proteins, most of which show reactivity based on homology to their rodent counterparts^12^. Concretely, 1,129 (1.1 k assay) and 1,310 (1.3 k assay) proteins were measured in CSF and plasma samples, respectively, from 6 MCAO and 6 sham-control animals. Animals were selected according to the total volume of CSF collected (minimum CSF volume required = 100 µl), and all samples were analyzed individually. Three different dilutions were performed to achieve all dynamic logs provided by the SOMAscan technology^75^. Normalization and calibration steps were then performed following SOMAscan technical instructions in order to remove systemic biases in the raw assay data. All samples passed SomaLogic quality control. Moreover, relative fluorescent unit (RFU) output from the array was subjected to background subtraction by establishing an accurate limit of detection threshold. To that end, a no-protein control sample was included in the assay run, which enabled the qualitative evaluation of baseline signal for each SOMAmer. Concretely, background subtraction discarded 216 (19.1%) and 29 (1.4%) proteins from the CSF and plasma datasets, respectively.

Comparisons between pre and post CSF samples were performed using paired t-test, corrected by the False Discovery Rate (FDR adjusted p-value), within each experimental group (R software 3.3.2, R development core team 2012, Austria, multtest R package) (Fig. 1B). Proteins presenting an FDR adjusted p-value < 0.05 were considered statistically significant. Base 2 logarithmic fold-changes (LogFC) were calculated for each protein by substracting abundance logarithmic values of the pre to the post CSF samples (LogFC = log [Post-CSF value/Pre-CSF value]). Comparisons on plasma samples were performed by using t-test (R software 3.3.2). Proteins presenting a p-value < 0.05 were considered statistically significant. Correlations between CSF and plasma levels were calculated using Pearson’s test.

### Bioinformatics analysis

All data on rat CSF were analyzed by Ingenuity Pathway Analysis (IPA, QIAGEN, USA). All proteins screened in the SOMAscan proteomic approach were introduced in the software. For each experimental group, a filter of FDR adjusted p-value < 0.05 for the comparison of proteins between pre and post CSF samples was applied. For the MCAO group, an additional cut-off of logFC > |1.5| was also used.

Data sets from MCAO and sham experimental groups were then characterized with the Ingenuity Pathways Knowledge Base, focusing the attention on the central nervous system and neuronal, immune and microvasculature cells types, and using all data sources (human, mouse and rat). A list of the main enriched biological processes, canonical pathways and molecular functions for each experimental data set were obtained according to the overlap p-values, calculated using right-tailed Fisher’s exact test corrected by the Benjamini-Hochberg multiple test (significant B-H p-value ≤ 0.05). Furthermore, both IPA analysis (MCAO and sham) were then compared side-by-side.

### Western Blot and Immunohistochemistry/Immunofluorescence

Seven protein candidates from the proteomics list (AREG, CaMK2A, CaMK2B, CaMK2D, CKB, CMPK and PDXP) were evaluated in rat brain protein homogenates and tissue sections by means of western blot (n = 4/8 shams and ischemic animals, respectively) and immunohistochemistry or immunofluorescence (n = 2 ischemic animals). Detailed protocols and procedures can be found in Supplemental materials and methods data.

### Human blood samples

All human studies were approved by the Ethics Committee of Vall d’Hebron Hospital (ischemic stroke -PR[AG]157/2011- and control - PR[IR]87/2010 - individuals) and written informed consent was obtained from all subjects or relatives in accordance with the Declaration of Helsinki. Ischemic stroke patients admitted to the emergency department of the Vall d’Hebron University Hospital (Barcelona, Spain) from December 2013 to November 2014 within the first 6 h after neurological symptoms onset. On admission, patients underwent a standardized protocol of brain imaging to differentially diagnose ischemic stroke. Trained neurologists assessed stroke severity using the National Institutes of Health Stroke Scale (NIHSS) and obtained demographic and clinical data from all patients. When eligible, ischemic stroke patients received the standard thrombolytic treatment (intravenous 0.9 mg/Kg recombinant tissue-plasminogen activator, rt-PA) and/or mechanical thrombectomy to remove the arterial clot. The clinical follow-up of each stroke patient was conducted at the 3rd month after stroke. At that time-point, functional outcome was evaluated according to the modified Rankin Scale (mRS); patients with a mRS score from 0 to 2 were classified as “good outcome” group and patients with a mRS from 3 to 6 as “poor outcome” group.

Blood samples from all patients were drawn on admission (<6 h from symptoms onset) and before administration of any treatment in EDTA tubes. Samples were then centrifuged at 1,500 g at 4 °C for 15 minutes and plasma was stored at −80 °C until further use. From this sample collection, a total of 38 ischemic stroke patients were selected for this study. Additionally, a total of 16 volunteers were included as controls.

### Human ELISA

Five protein candidates (CaMK2A, CaMK2B, CaMK2D, CKB and CMPK) were further evaluated in blood samples from the aforementioned ischemic stroke patients and controls using commercially available ELISA kits. Detailed methods are available in Supplemental materials and methods data.

### Statistical analyses

SPSS statistical package 22.0 was used for statistical analyses and GraphPad Prism 6.0 for creating graphs. Rat brain and human plasma protein data distribution was assessed by Shapiro-Wilk and Kolmogorov-Smirnov test, respectively. To determine differences in protein levels between experimental groups, Student t-test (normally distributed variables) or Mann-Whitney (non-normally distributed variables) were used. To analyze differences on clinical variables among patients, continuous factors were analyzed by Student’s test (normally distributed, mean and SD values) or Mann-Whitney test (non-normally distributed, median and interquartile range (IQR)), while categorical variables were assessed by Pearson chi-squared test (frequencies). Correlations between protein levels and clinical continuous variables were analyzed using Pearson’s test (normally distributed variables) or Spearman’s test (non-normally distributed variables).

Receiver operating characteristics (ROC) curves were used to obtain the cut-off points of circulating protein levels with optimal accuracy (both sensitivity and specificity) for discriminating ischemic stroke prognosis. Forward stepwise multivariate logistic regression analysis for poor functional outcome was performed with all clinical variables associated with this endpoint at p < 0.1. Odds ratio (OR) and 95% confidence interval (CI) were adjusted by sex, age and NIHSS score at admission. Using the previous identified cut-off points, baseline levels of each associated prognostic biomarker were added to the clinical model, singly or in combination, to assess their independent association and to build new predictive models.

P-value < 0.05 was considered significant at a 95% confidence level in all cases.

### Data availability

The datasets generated during and/or analyzed during the current study are available from the corresponding author on reasonable request.

## Electronic supplementary material

Supplementary material and methods

Supplementary table S1
