# Supplementary material for: Characterization of the rat cerebrospinal fluid proteome following acute cerebral ischemia using an aptamer-based proteomic technology
Source: Sci Rep. 2018 May 21;8:7899. doi: 10.1038/s41598-018-26237-3 (PMC5962600; doi:10.1038/s41598-018-26237-3)
Supplement: Supplementary file 1 — Supplementary material and methods [file 41598_2018_26237_MOESM1_ESM.pdf]

Characterization of the rat cerebrospinal fluid proteome following acute cerebral ischemia using an aptamer-based proteomic technology

Alba Simats, Teresa García-Berrocoso, Laura Ramiro, Dolors Giralt, Natalia Gill, Anna Penalba, Alejandro Bustamante, Anna Rosell, Joan Montaner

SUPPLEMENTARY MATERIAL

SUPPLEMENTARY MATERIAL

- Supplementary methods
- Supplementary tables
- Supplementary figures

SUPPLEMENTARY METHODS

Western Blot

Top protein candidates from the proteomics list were chosen to be evaluated in rat brain protein homogenates from MCAO and sham-control animals (n=8/4 per group, respectively). Equal protein amounts of 25 µg were resolved in 12% sodium dodecyl sulfate-polyacrylamide gels under reducing conditions and transferred onto nitrocellulose membranes (Amersham Bioscience, Amersham, UK). Membranes were blocked with 10% non-fat milk and the following primary antibodies were incubated: Rabbit anti-amphiregulin (AREG) (1/1,000) (Bioss Antibodies, USA), mouse anti-calcium/calmodulin-dependent protein kinase II subunit alpha (CaMK2A) (1/10,000) (ThermoFisher Scientific Inc.), mouse anti-calcium/calmodulin-dependent protein kinase II subunit beta (CaMK2B) (1/5,000) (ThermoFisher Scientific Inc.), rabbit anti-calcium/calmodulin-dependent protein kinase II subunit delta (CaMK2D) (1/5,000) (Proteintech Group Inc., Rosemont, USA), rabbit anti-calcium/calmodulin-dependent protein kinase II (CaMK2, total) (1/1,000) (Abcam, Cambridge, UK), rabbit anti-T-286 phosphorylated calcium/calmodulin-dependent protein kinase II (CaMK2, phospho-T286) (1/10,000) (Abcam), mouse anti-creatine kinase B-type (CKB) (1/5,000) (Abcam), rabbit anti-uridine monophosphate/cytidine monophosphate (UMP/CMP) kinase (CMPK) (1/1,000) (Proteintech Group Inc.), goat anti-pyridoxal phosphate phosphatase (PDXP) (0.1µg/ml) (Novus Biologicals, Littleton, USA) and anti-β-actin (1/500) (Sigma Aldrich Inc., MO, USA). Membranes were then incubated with secondary antibodies linked to horseradish peroxidase (HRP) (ThermoFisher Scientific Inc.) (anti-rabbit HRP (1/1,000), anti-mouse HRP (1/1,000) or anti-goat HRP (1/5,000)) with gentle agitation. Finally, substrate reaction was developed with chemiluminescent reagent Luminol (Amersham Biosciences) and analyzed with Odissey Fc Imaging System (Li-Cor, USA). Western blots were then quantified using Image-J free software. All positive signals were corrected by β-actin signal, used as loading control. Afterwards, IP quantified bands were normalized by their respective CL bands and statistical comparison was performed between the IP/CL ratios of the experimental groups.

Immunohistochemistry and immunofluorescence

Selected candidates were evaluated by means of immunohistochemistry (IHC) or immunofluorescence (IF) in paraffin-embedded rat brain slides (n=2, MCAO animals). In brief, brain sections were heated at 65ºC and were subjected to a standard deparaffinization procedure with a final wash in 0.1% Tween-Tris buffered saline (TBST). Subsequently, all brain sections were submitted to antigen retrieval using citrate buffer (pH 6, 95º), allowed to temper and blocked with 10% goat serum (Millipore Corporation, MA, USA) in TBST for 1h.

For IHC purposes, slides were then incubated with the aforementioned primary anti-rat antibodies in the following conditions: AREG (1/500), CaMK2B (1/250), CaMK2D (1/250), CMPK (1/100) and CKB (1/100). Then, slides were incubated with the corresponding biotinylated secondary antibody against IgG (1/250) (Vector Laboratories Inc., Burlingame, CA, USA), and consecutively with HRP-streptavidin (1/100) (Vector Laboratories Inc.). Finally, brain sections were submerged to Liquid DAB+ (diaminobenzidine) (Dako, Carpinteria, CA, USA) and stained with Harris hematoxylin (Sigma Aldrich Inc.) and mounted on coverslips using DPX mounting medium (Sigma Aldrich Inc.).

For IF, slides were incubated with the primary anti-rat antibodies against CKB (1/100), Glial Fibrillary acidic protein (GFAP) (1/200) (ThermoFisher Scientific Inc.) and Ionized calcium binding adaptor molecule 1 (Iba-1) (1/750) (Abcam) followed by AlexaFluor 488 anti-rabbit IgG and AlexaFluor 568 anti-mouse IgG (Life Technologies, USA) respectively, and with 0.3% Sudan Black B (Sigma Aldrich Inc.) to reduce brain tissue autofluorescence [3]. Sections were mounted on coverslips using Vectashield with 46-diamidino-2-phenyl indole (DAPI; Vector laboratories).

In all cases, negative controls were performed without applying the primary antibody. All brain sections were analyzed using an Olympus BX61 microscope (Olympus, Japan) and the same intensities and parameters were set for all slides. Images were processed with the Olympus CellSens Imaging software (Olympus) and overlaying fluorescent images were created with Image J free software.

Human ELISA

Outstanding candidates from the animal study were evaluated in a pilot study with blood samples from ischemic stroke patients and controls using commercially available ELISA kits. Circulating levels of CaMK2A (Cat.# DL-CAMK2a- Hu, DLdevelop, China), CaMK2B (Cat.#E01C1335, BlueGene BioTech Co., China), CaMK2D (Cat.#E01C1336, BlueGene BioTech Co.), CKB (Cat.#E-EL-H2433, Elabscience Biotechnology Co., China) and CMPK (Cat.#E14030h, Wuhan EIAab Science Co., Ltd, China) were assessed following manufacturer's instructions. Each candidate was evaluated in all 38 samples from ischemic stroke patients, and in 8 randomly-selected samples from the 16 selected volunteers (controls). Each sample was assayed per duplicate and the mean value was used. Optical densities (OD) were measured in a Synergy TM Mx microplate reader (BioTek Instruments Inc, USA). Samples with a coefficient of variation (CV) higher than 20% were excluded for the analysis. Standard curves from each plate were used as inter-assay controls for each commercial kit. When values were under the detectable range of the assay, the limit of detection of the assay value was assigned to them. When inter-assay CV was higher than 20%, values were standardized prior to statistical analysis by calculating the Z-score value by dividing the mean of each 96-well plate kit by the standard deviation and adding two units to avoid results below zero in any sample.

SUPPLEMENTARY TABLES

Factors	Ischemic strokes (n=38)	Healthy controls (n=16)	p-value
Age (years)	76.55 ±10.02	68.47 ±5.18	**0.016**
Sex(Male)	17 (44.7%)	8 (50%)	0.723
Admission NIHSS score	10.34 ± 6.33	-	-
Smokers	4 (12.1%)	2 (25%)	0.578
Alcohol consumption	3 (7.9%)	2 (25%)	0.268
Arterial hypertension	29 (76.3%)	10 (62.5%)	0.333
Diabetes mellitus	10 (26.3%)	2 (12.5%)	0.229
Dyslipidemia	15 (39.5%)	4 (25%)	0.309
Atrial Fibrillation	20 (52.6%)	0 (0%)	**<0.001**
Ischemic cardiopathy	17 (44.7%)	2 (12.5%)	**0.024**
Coronary artery disease	4 (10.3%)	1 (6.3%)	0.923
Previous stroke	6 (15.8%)	0 (0%)	0.163
Acute treatment	22 (57.9%)	-	-

Table S2. Demographic and clinical factors of ischemic stroke patients and healthy controls. Age and NIHSS score at admission are expressed as mean ± SD and all other clinical variables are expressed as frequency of patients that present the specified condition. Statistically significant p-values are highlighted in bold. Abbreviations: NIHSS: National Institutes of Health stroke scale.

Factors	3-month outcome		
	Good outcome (n=17)	Poor outcome (n=21)	p-value
Age, years	79 (74-82)	81 (69-83)	0.45
Gender (Male)	8 (47.1%)	9 (42.9%)	0.796
Admission NIHSS score	10 (4-11)	11 (5-17)	0.186
Smokers	3 (21.4%)	1 (5.3%)	0.288
Arterial hypertension	13 (76.5%)	16 (76.2%)	1
SBP, mm Hg	148 (140-155)	142 (115.5-167.5)	0.304
DBP, mm Hg	80 (74.5-82)	74 (62.5-85)	0.685
Glycemia, mg/dL	110 (102.5-133)	142 (129-176.5)	**0.02**
Diabetes mellitus	4 (23.5%)	6 (28.6%)	1
Dyslipidemia	8 (47.1%)	7 (33.3%)	0.389
Atrial Fibrillation	7 (41.2%)	13 (61.9%)	0.203
Ischemic cardiopathy	6 (35.3%)	11 (52.4%)	0.292
Coronary artery disease	0 (0%)	4 (19%)	0.113
Previous stroke	4 (23.5%)	2 (9.5%)	0.378
Acute treatment	12 (54.5%)	10 (45.5%)	0.233
TOAST			0.766
- Atherothrombotic	5 (29.4%)	3 (14.3%)	
- Cardioembolic	7 (41.2%)	14 (66.7%)	
- Lacunar	1 (5.9%)	1 (4.8%)	
- Undetermined	4 (23.5%)	3 (14.3%)	

Table S3. Univariate analysis. Clinical characteristics and factors associated with outcome at third month after stroke. Statistical significant differences between groups are expressed as bold p-value. Abbreviations: NIHSS: National Institutes of Health stroke scale; SBP: systolic blood pressure; DBP: diastolic blood pressure; TOAST: etiology stroke subtype classification.

SUPPLEMENTARY FIGURES

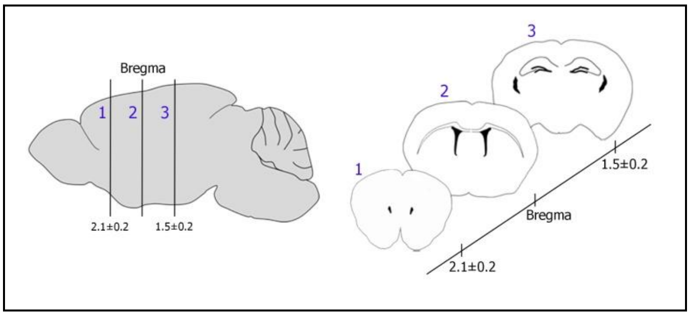

Figure S1. Representative illustration of the three cortical depths in which brains were cut for immunostaining purposes: ***1*** indicates 2.1 ± 0.2 mm anterior to bregma, ***2*** indicates the bregma point and ***3*** indicates 1.5 ± 0.2 posterior to bregma.

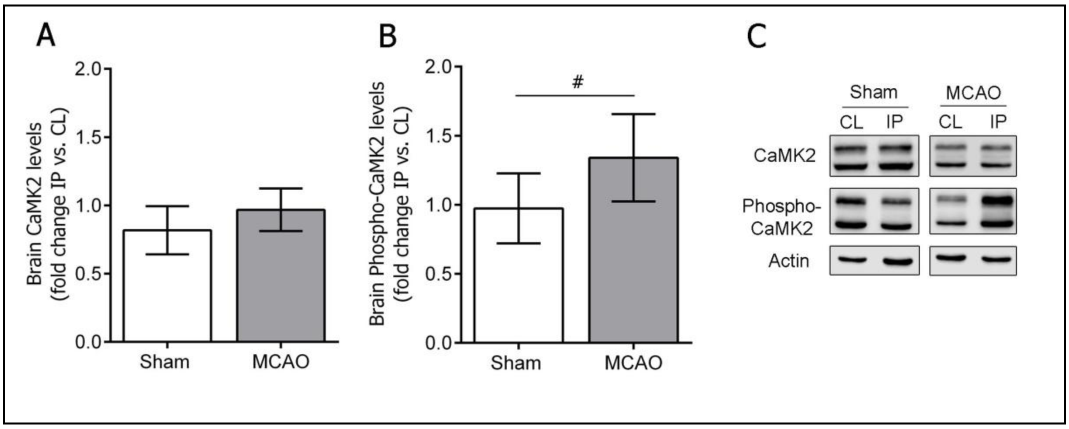

Figure S2. CaMK2 and phosphorylated CaMK2 protein levels in the brains of ischemic and sham-control animals. Plot of (**A**) total CaMK2 and (**B**) phosphorylated CaMK2 protein abundances (relative quantification, ratio IP vs. CL hemisphere of each animal, both run in the same blot) in brain homogenates samples obtained 2h after MCAO or sham-control surgery (n=4 samples for sham; n=8 samples for MCAO). (**C**) Representative Western Blots signals of the two detected bands for each used antibody. # indicates p<0.1. Abbreviations: IP: ipsilateral hemisphere; CL: contralateral hemisphere.

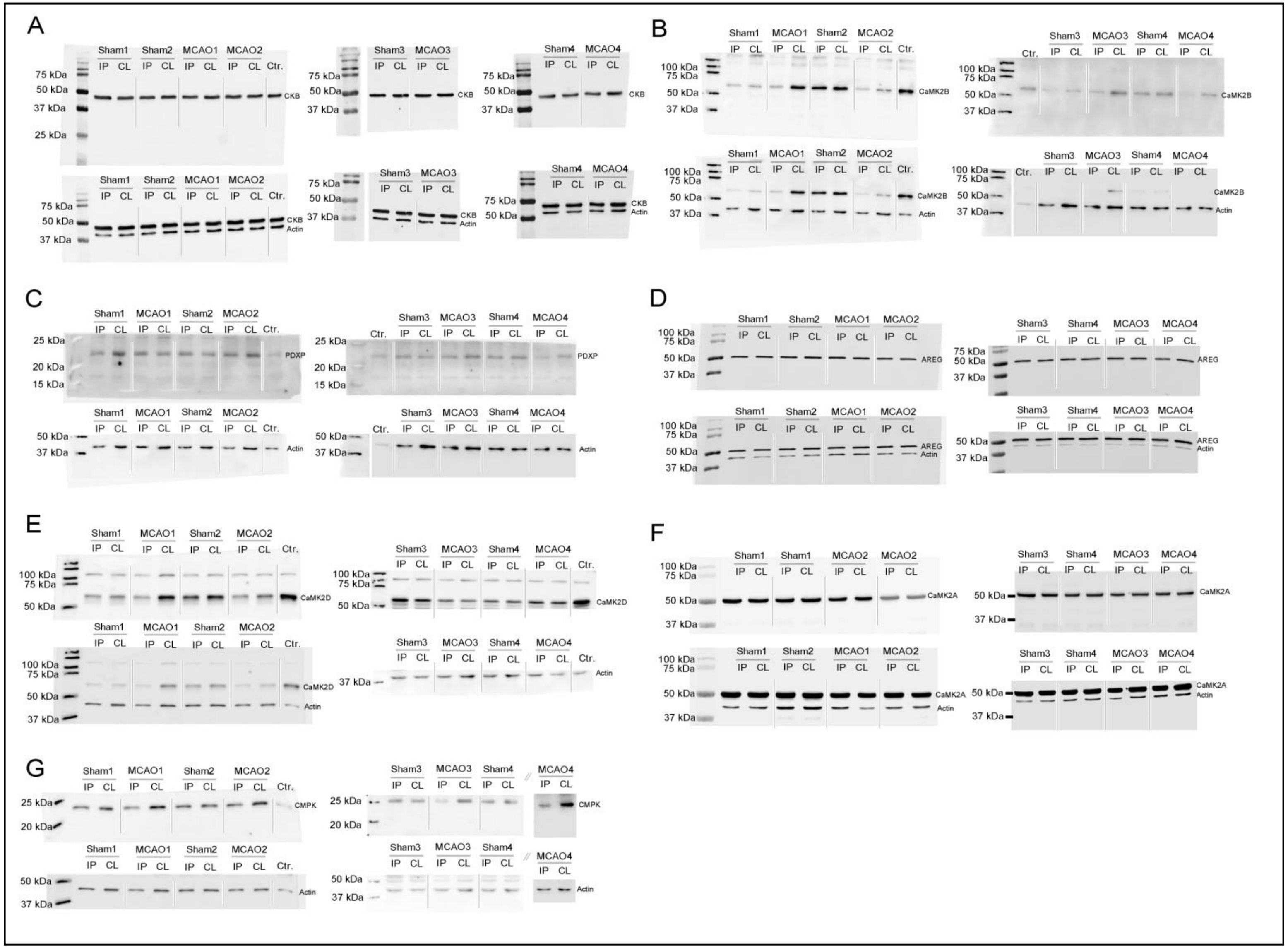

Figure S3. **Illustrative images of the full-length blots.** (**A**) CKB, (**B**) CaMK2B, (**C**) PDXP, (**D**) AREG, (**E**) CaMK2D, (**F**) CaMK2A and (**G**) CMPK. Each panel (A-G) illustrates IP and CL samples of four different sham and ischemic animals. The band corresponding to the protein under consideration (top) and the signal of β-actin (bottom) are shown for each sample. In all cases, IP and CL samples from each animal are run in the same gel and individually corrected by their respective β-actin loading controls.
